# Cyclin D/CDK4/6 activity controls G1 length in mammalian cells

**DOI:** 10.1371/journal.pone.0185637

**Published:** 2018-01-08

**Authors:** Peng Dong, Carolyn Zhang, Bao-Tran Parker, Lingchong You, Bernard Mathey-Prevot

**Affiliations:** 1 Department of Pharmacology and Cancer Biology, Duke University School of Medicine, Durham, NC, United States of America; 2 Department of Biomedical Engineering, Duke University, Durham, NC, United States of America; 3 Center for Genomic and Computational Biology, Duke University, Durham, NC, United States of America; 4 Department of Molecular Genetics and Microbiology, Duke University School of Medicine, Durham, NC, United States of America; 5 Department of Pediatrics, Duke University School of Medicine, Durham, NC, United States of America; Georgetown University, UNITED STATES

## Abstract

The length of the G1 phase in the cell cycle shows significant variability in different cell types and tissue types. To gain insights into the control of G1 length, we generated an E2F activity reporter that captures free E2F activity after dissociation from Rb sequestration and followed its kinetics of activation at the single-cell level, in real time. Our results demonstrate that its activity is precisely coordinated with S phase progression. Quantitative analysis indicates that there is a pre-S phase delay between E2F transcriptional dynamic and activity dynamics. This delay is variable among different cell types and is strongly modulated by the cyclin D/CDK4/6 complex activity through Rb phosphorylation. Our findings suggest that the main function of this complex is to regulate the appropriate timing of G1 length.

## Introduction

During the gap 1 (G1) phase of the cell cycle, cells grow in size and synthesize mRNAs and proteins in preparation for the S phase when DNA is replicated [1,2]. During this phase, cells integrate signals from growth stimuli, nutrient supplies and differentiation cues to determine whether conditions are met to proliferate [3,4]. G1 differs from other cell cycle phases in that its duration is highly variable among different cell types. Indeed, its length is the primary determinant in the variation of the whole cell cycle timespan [5,6]. Embryonic stem cells have extremely short G1 phases, and lengthening of this phase is a hallmark of lineage specification when stem cells differentiate into tissue-specific progenitor cells [5,7,8]. In the hematopoietic system, the coupling between cell-cycle lengthening and transcriptional regulation determines lymphoid and myeloid differentiation [9]. Moreover, the G1 length has been speculated to influence the differentiation of embryonic neural stem cells and long-term hematopoietic stem cells [7,10]. Conversely, an ultrafast cell cycle with barely any existing G1 phase was shown to correlate with over 99% of the bulk reprogramming activity in fibroblast cells induced to become induced pluripotent stem cells (iPSC), confirming a critical relationship between cell cycle length and cell fate determination [11]. Therefore, clarifying the mechanisms that modulate G1 duration is likely to provide key insights into understanding a number of biological processes such as embryogenesis and tissue homeostasis.

Despite our extensive knowledge of the cell cycle regulation, how the known molecular effectors control G1 length in single cells remains poorly understood. In the canonical model for cell cycle regulation, the control of the G1/S transition mediated by the Rb/E2F network is thought to be exerted by multiple G1 cyclin/cyclin-dependent kinase (CDK) complexes [12]. Furthermore, it has been proposed that a fine-tuning of G1 cyclin/CDK activities by upstream signaling pathways would achieve an ordered G1 progression [13].

Analysis of cell-cycle progress in cell populations is insufficient to address this issue, given the extensive cell-cell heterogeneity in cellular behavior and gene expression [14–16]. In contrast, single-cell analysis represents a powerful approach to develop quantitative understanding of how the gene regulatory network encodes and decodes dynamic signals to coordinate cell-cycle progression [17]. A recent study reported that, at the single cell level, the cell-cycle commitment in G1 tightly correlates with APC^*Cdh1*^ inactivation shortly before S phase entry and significantly later than E2F target gene expression, suggesting that more intricate regulation of E2F activity at multiple layers may exist at the temporal scale [18]. Moreover, it remains unexplored how the regulation of E2F activity is coordinated with the progression of G1 phase.

To this end, we developed an E2F activity reporter that captures the dynamic regulation of free E2F activators following their release from the inhibitory sequestration by Rb. We used this reporter to monitor E2F activity dynamics at the single-cell level and in real time. Our results indicate that the E2F activity reporter serves as a reliable indicator of S phase entry at the temporal scale. Furthermore, our quantitative analysis of E2F activity dynamics linked to G1/S transition underscores the role of cyclin D/CDK4/6 activity as a main determinant of G1 length.

## Material and methods

### E2F1 transcriptional and activity reporters

A clone of REF52 cells expressing the transcriptional reporter pQCXIP-hE2F1p::4NLS-d4Venus was described in our previous publication [19]. For the activity reporter, a DNA fragment encoding dsmCherry (mCherry fused with a PEST degradation tag at the C terminus, ~ 2 hrs half-life) was fused with an SV40 nuclear localization sequence (NLS) at its N-terminus to generate the NLS-dsmCherry-expressing cassette. This cassette was then subcloned after a truncated human E2F1 promoter (-150 ~ +32 bps) similar to the fragment used by Araki et al. [20] into a pQCXIP vector (Clontech) to construct the pQCXIP-shE2F1p::NLS-dsmCherry reporter plasmid. The derived construct was transfected into an ecotropic packaging cell line, Plat-E. Forty-eight hours after transfection, the culture medium containing retrovirus particles was filtered and applied to REF52 cells. Positive cells genetically integrated with the reporter system were then sorted by FACS. Single clones (REF52-shE2F1p::NLS-dsmCherry) were obtained from sorted cells after limiting dilution and expansion of single cells plated in a 96-well plate. To construct the shE2F1p::NLS-dsmCherry/ hE2F1p::4NLS-d4Venus dual reporter REF52 cells, virus packaged from pQCXIP-shE2F1p::NLS-dsmCherry reporter plasmid was applied to the previously generated REF52-hE2F1p::4NLS-d4Venus E2F transcriptional reporter REF52 cells and sorted for positive clones. Single colonies were isolated as above. To generate the shE2F1p::NLS-dsmCherry/Geminin-GFP dual reporter REF52 cells, pQCXIP-Geminin-GFP plasmid (a gift from Igor Shats, Duke University) was packaged into virus and infected REF52-shE2F1p::NLS-dsmCherry cells. Similar procedures were used to generate hTert-HME1 cells integrated with E2F transcriptional and activity reporters, with the difference that an amphotropic packaging cell line, Plat-A, was used to produce virus stocks.

### Cell culture

REF52 (an immortal line of post-crisis Fischer rat embryo cells) fibroblasts were routinely grown in Minimum Essential Medium Alpha Medium (Gibco/Invitrogen) supplemented with 10% bovine growth medium (BGS, Hyclone/Thermo Scientific). hTert-HME1 (CRL-4010, ATCC) cells were cultured in Mammary Epithelial Basal Medium (MEBM, Lonza, CC-3151) with addition of MEGM SingleQuots (without addition of GA-1000 component, Lonza, CC-4136). For the preparation of E2F dynamics measurement by using time-lapse microscopy, cells were first trypsinized, resuspended at a density of 1×10^5^ per ml and then seeded in μ-Slide I (tissue culture treated, ibidi) channel slides by adding 1 ml volume of the cell suspension. After overnight culturing, REF52 cells were synchronized in quiescence by shifting into Minimum Essential Medium Alpha medium with 0.02% BGS (starvation medium) for 36 h. For perturbation experiments, PD0332991 (CDK4/6 inhibitor, ChemieTek) was added into cells immediately after cells were released from serum starvation (by adding 10% BGS). hTert-HME1 was starved in basal medium (MEBM without additive) for 36 hours after seeding overnight.

### Live cell imaging

For time-lapse microscopy, quiescent cells growing in μ-Slide I slides were released from starvation by shifting to serum containing medium and placed under Leica DMI 6000 B inverted fluorescence microscope (Leica). Images were taken using Leica N PLAN L 20×0.4 objective lens with phase contrast, Semrock BrightLine GFP filter set, YFP filter set or Texas Red filter set, and Hamamatsu ORCA AG digital camera (Hamamatsu) with uniform parameter setting: binning = 4, offset = 0, gain = 255 and exposure time = 0.01 s (phase channel) or 0.15 s (YFP channel). The microscope was enclosed with an environmental chamber with 37 C temperature, atmosphere (5% CO2) and humidity. Images were acquired every 30 or 60 min for 24~48 h. Time-series image acquisition was controlled by SimplePCI 6 Software (Hamamatsu).

### Immunostaining, EdU staining and imaging

Immunostaining was performed after E2F dynamics measurement in ibidi μ-Slide I slides with Phospho-Rb (Ser807/811) (D20B12) XP Rabbit mAb (Alexa Fluor 488 Conjugate, Cell Signaling) following the manufacturer’s instruction. EdU staining was performed after E2F dynamics measurement in ibidi μ-Slide I slides by using Click-iT EdU Imaging Kits (Invitrogen) with Alexa Fluro 594 azide according the manufacturer’s instructions. The only modification of the protocols for immunostaining and EdU staining was to wash cells with PBS with 0.2% Tween three times after all the staining steps. Images were taken using Leica N PLAN L 20×0.4 objective lens with phase contrast or a Semrock BrightLine GFP filter set, YFP filter set, Texas Red filter set, and Hamamatsu ORCA AG digital camera with uniform parameter setting: binning = 4, offset = 0, gain = 255 and exposure time = 0.01 s (phase channel) or 0.8 s (Red channel).

### Image analysis

The time-lapse microscopy resulted in two series of raw images of the cells in 30 or 60-min increments for 36–72 h, one each for the phase channel and the fluorescence channel(s). E2F signals were extracted from these images using ImageJ (NIH) software. The first time-point of each series of images was loaded side-by-side into the software. Using the ROI Manager Tool, a circular selective marker with a fixed area was placed around each cell nucleus in the phase channel image. The location of each marker was then copied to the fluorescence channel(s) image and the integrated grey value of the selected pixels was measured. In the case of cell division, the selective marker was applied to one of the daughter cells. This value was normalized to the background by subtracting the integrated grey value of the same area of pixels in an empty part of the image. This process of measuring normalized grey value of the nuclei was repeated for each time point by adjusting the location of the selective marker to account for the movement of cells, thus giving a time-series measurement of the fluorescence reporter on the E2F fluorescence. Throughout this process, data from cells that left the field of view at any time were discarded; and the time of cell division was also noted. The similar approach was applied to quantify the fluorescence signal from immunostaining and EdU staining.

### Analysis of E2F dynamics trajectories

This time-series E2F dynamics trajectories were analyzed in Matlab (MathWorks) to determine various E2F parameters, e.g. time delay, amplitude, etc. The processed trajectories without spikes were then smoothened by using a 3-window Gaussian averaging algorithm. Each smoothed and processed trajectory was then fit to the following two-phase regression model to automatically derive optimal values for *t*_1_ and *t*_2_:

$$y=\{\begin{array}{c} y_{0},0\leq t<t_{1}\\ (y_{\max}-y_{0})\cdot (t-t_{1})/t_{2},t_{1}\leq t\leq t_{2} \end{array}$$

Thus, the problem equals to search for arg {*t*_1_,*t*_2_} that gives $\min\left\{\sum_{0\leq t\leq t_{2}}{\left[y_{E2F}(t)-y(t)\right]}^{2}\right\}$.

Relevant parameters represent:

*t*_1_: initial delay
*t*_2_: activation time
*y*_0_: basal level (the average of fluorescence values of the initial four time points)
*y*_max_: peak level (maximum fluorescence value of each trajectory)
*y*_E2F_(*t*): E2F signal in dynamic trajectory at the moment *t*

### Modeling and simulation analysis

**Fig 1:**
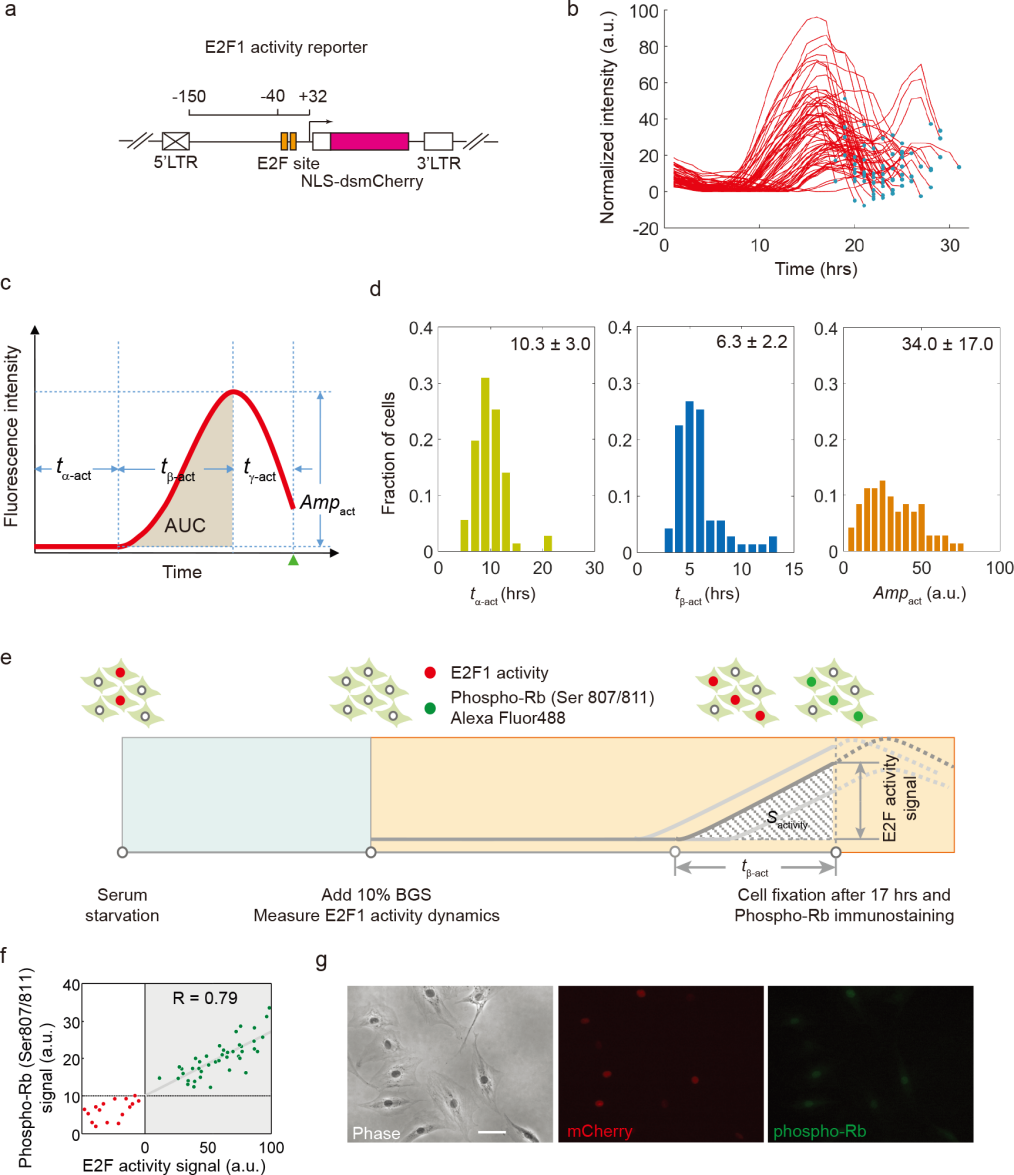
Measurement of E2F activity dynamics at the single-cell level. (a) The schematic of E2F1 activity reporter. A minimal promoter sequence of human E2F1 (-150 ~ +32) without E-box (Myc binding site) was cloned before the destabilized mCherry coding sequence. (b) Trajectories of E2F1 activity in single REF52 cells released from serum starvation. The dot in each trajectory corresponds to the cell division time point. (c) Metrics to quantify E2F activity: *t*_*α*_*-*act, initial delay; *t*_ß-act_, activation time; *t*_*γ*-act_, post-activation time; *Amp*_act_, amplitude; *AUC*, area under curve for E2F1 activity increase. Green triangle, cell division time point. (d) Distributions of *t*_*α*-act_, *t*_ß-act_ and *Amp*_act_ of about 70 cells. (e) Experimental design to align E2F1 activity to Rb phosphorylation. REF52 cells integrated with E2F1 activity reporter were released from serum starvation and the reporter signal was measured every 30 minutes. At 17 hours, when most cells enter S phase, cells were fixed and immunostained with antibody against phosphorylated Rb protein conjugated with Alexa Fluo 488. (f) Correlation between the E2F1 activity signal (mCherry) and Rb phosphorylation at the time of fixation for each individual cell. Green dots indicate cells with an increase in E2F1 activity (activated) and red ones indicate cells basal E2F1 activity (inactivated) at 17 hours. Green dots were fitted to a linear model (R = 0.79). (g) Representative views of cells in imaged in phase, mCherry and Alexa Fluo 488 (phosphorylated Rb) channels. Scale bar, 50 μm.

The equations defined in S1 and S2 Tables, along with the corresponding parameters in S3 Table are utilized to simulate the temporal dynamics of E2F activity and transcription in Matlab (MathWorks). To compare E2F transcriptional and activity dynamics regulated by wild-type network or the network with Rb deletion, we simulated models in either S1 Table (wild-type) or S2 Table (Rb deletion) and plot the trajectories in the corresponding results section. To examine the effects of cyclin D/CDK4/6 inhibition and cyclin E/CDK2 inhibition on *t*_*Δ*_, we modify the decay constant of cyclin D, *d*_CD_, and cyclin E, *d*_CE_, respectively. In both cases, we change the decay constant between 1.5/h– 4.5/h. We demonstrate the effect of inhibiting either cyclin E/CDK2 or cyclin D/CDK4/6 under these conditions on *t*_Δ_, E2F activity, and E2F transcription (see relevant results section). The definition of *t*_Δ_ is provided in the text under the appropriate figure, and both E2F activity and transcription are measured in terms of the AUC as defined by Fig 1C.

### Statistical analysis

*t*_*Δ*_ is calculated from measured trajectories in both control (DMSO) and perturbed (1 μM PD0332991) conditions. The statistics are reported in both box and beeswarm plots and the difference is estimated by Wilcoxon rank test.

## Results and discussion

### Measurement of E2F activity dynamics at the single-cell level

E2Fs are a family of transcription factors whose activities are directly linked to cell cycle control. The E2F family comprises both transcriptional activators (E2F1-3a) and repressors (E2F3b-8). E2F activators are regulated at multiple levels, with a key regulation of their activity provided via their tight but reversible sequestration by the retinoblastoma (Rb) tumor suppressor protein [13,21,22]. It is widely accepted that only “free” E2F activators dissociated from Rb can initialize the G_1_/S transcriptional program leading to cell cycle entry. To measure the regulatory activity of free E2F protein (henceforth referred as “E2F activity dynamics”) in individual cells, we engineered an E2F activity reporter by placing a destabilized mCherry protein downstream of a truncated human E2F1 promoter. The truncated E2F1 promoter only includes the two putative E2F binding sites, but lacks the canonical Myc binding site and other potential regulatory sites located in the more distal region of the E2F1 promoter (Fig 1A). A similar E2F1 promoter fragment was shown to promote increased transcription of genes under its control in response to mitogenic signals, in an E2F-dependent manner [20]. Following stable integration of this construct into rat REF52 fibroblasts, we derived independent cell clones expressing the activity reporter.

We next examined the behavior and activity dynamics of this reporter system in real time in single REF52 rat fibroblasts held in G0 through serum starvation and released into the cell cycle by serum stimulation. We quantified the level of fluorescence over time in individual cells throughout the first cell cycle (S1 Movie). Although the reporter activity tracings were highly variable between cells, they all followed the same temporal pattern: after an initial delay (6-20hrs), the activity in each cell gradually increased to a maximum and then decreased by 20–60% before cell division (Fig 1(panels b and c), S1 Fig (panel a)). The activity in several cells stayed around the baseline; these cells did not divide during the time of the experiment (S1 Fig (panel a)). The temporal pattern of E2F activity dynamics was similar in hTert-HME1 human mammary epithelial cells stably integrated with the E2F activity reporter (S1 Fig (panel b)). This temporal pattern can be captured by several metrics, including the maximum amplitude (*Amp*_act_), the initial delay (*t*_α-act_), the activation time (*t*_ß-act_), and, for cells that undergo division, a post-activation time (*t*_*γ*-act_). The area under the curve (AUC) of the activity tracing, a metric geometrically related to the previous variables, captures the integrated activity of E2F over time (Fig 1C). As shown in Fig 1D, each metric was highly variable between cells. This variability was likely due to the heterogeneity in gene expression in individual cells during cell cycle progression (Fig 1D and S2 Fig). We reasoned that the increase in E2F activity would be the direct consequence of free E2F proteins released from Rb sequestration due to increased levels of phosphorylation by the G1 cyclins in complex with their respective cyclin dependent kinases (CDKs). Consistent with this notion, we found that the E2F1 activity was linearly correlated (R = 0.79) with the amount of phosphorylated Rb, measured by immunostaining (Fig 1E–1G). This correlation was maintained in hTert-HME1 stably integrated with the E2F activity reporter (S3 Fig). These data confirm that our reporter is a reliable proxy for monitoring E2F activity in single cells.

### The integral of E2F activity correlates with DNA synthesis at the temporal scale

**Fig 2:**
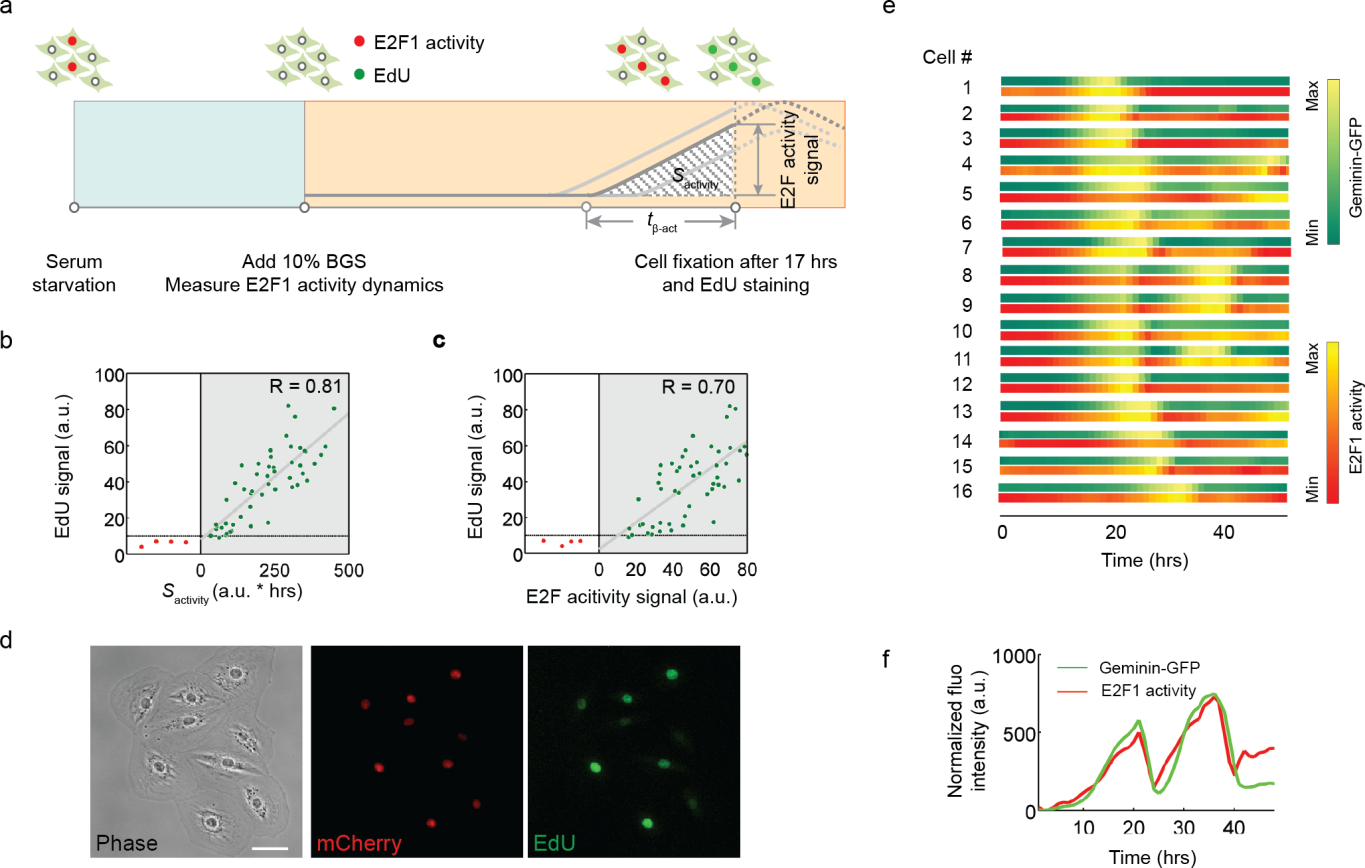
The integral of E2F activity correlates with DNA synthesis at the temporal scale. (a) Experimental design to relate E2F1 activity with DNA synthesis. REF52 cells expressing the E2F1 activity reporter were released from serum starvation and cultured in medium with 10% BGS and EdU. Images were taken every 30 minutes. Cells were fixed and stained for EdU labeling (Alexa Fluo 488) at 17 hours, when most cells enter S phase. (b) Correlation between the EdU staining signal and the corresponding integrated E2F1 activity (*S*_activity_). Green dots indicate cells with an increase in E2F1 activity (activated) and red dots indicate cells with basal E2F1 activity (inactivated) at 17 hours. Green dots were fitted to a linear model (R = 0.81). (c) Correlation between the EdU staining signal and the E2F1 activity signal measured at t = 17h. Green dots were fitted to a linear model (R = 0.70). (d) A representative view of cells as imaged in phase, mCherry and Alexa Fluo 488 (EdU) channels. (e) Temporal correlation between Geminin-GFP dynamics (GFP) and E2F1 activity dynamics (mCherry) in 15 individual cells during cell cycle progression for 55 hours. Fluorescence intensity was linearly normalized within the dynamics range of each cell. (f) An example of aligned Geminin-GFP dynamics and E2F1 activity dynamics trajectories (48 hours).

The release of E2F proteins from Rb sequestration is critical for initiating the transcription of numerous genes that participate in DNA synthesis, cell metabolism and growth and readies a cell for S phase entry. As the amount of DNA increases during DNA replication, we tested whether that amount would be correlated with the integrated E2F activity over time, or with the E2F activity at a specific time point (e.g. the end of the observation time). To this end, we combined the measurements of E2F activity with that of 5-ethynyl-2’-deoxyuridine (EdU) incorporation into newly synthesized DNA, a surrogate for determining how much DNA has been synthesized over time (Fig 2A). Our measurements indicate that the DNA synthesis was more strongly correlated with the E2F activity integrated over time (R = 0.81, Fig 2B) than with the E2F activity at the time of cell fixation (R = 0.70, Fig 2C and 2D). A similar observation was also made in hTert-HME1 cells (S4 Fig).

To measure the onset time of E2F activity relative to the start of S phase, we constitutively expressed a GFP-PCNA fusion protein [23] in REF52 cells expressing the E2F activity reporter. Live imaging of single cells induced back into the cell cycle with the addition of serum showed that the onset of E2F activity preceded the appearance of fine puncta for GFP-PCNA staining in the nucleus by 60–90 min, a stereotypic pattern observed in early S phase [24] in the majority of cells under observation (S5 Fig).

To further examine the temporal correlation between E2F activity dynamics and S phase entry, we relied on the property of the FUCCI reporter system [25]. During cell cycle progression, the signal from the proteolysis-regulated Geminin reporter starts to accumulate in S phase and sharply decreases at the end of M phase. We therefore decided to integrate the Geminin-GFP reporter into cells that contained the E2F activity reporter to generate a dual reporter system. The alignment of mCherry and GFP signals in individual cells indicates that the dynamics of both reporters are highly correlated over time (Fig 2 (panels e, f) and S2 Movie), suggesting that E2F activity accumulation is precisely coordinated with S phase progression presumably through E2F-induced expression of rate-limiting target genes for DNA synthesis. Moreover, our results indicate that the initial delay (*t*_*α*-act_) in E2F activity dynamics is a good predictor of the length of the G1 phase, and that the mean *t*_α-act_ can vary substantially among different cell types (e.g., REF-52 fibroblasts vs. hTert-HME-1 breast epithelial cells).

### Temporal delay exists between E2F transcriptional and activity dynamics

**Fig 3:**
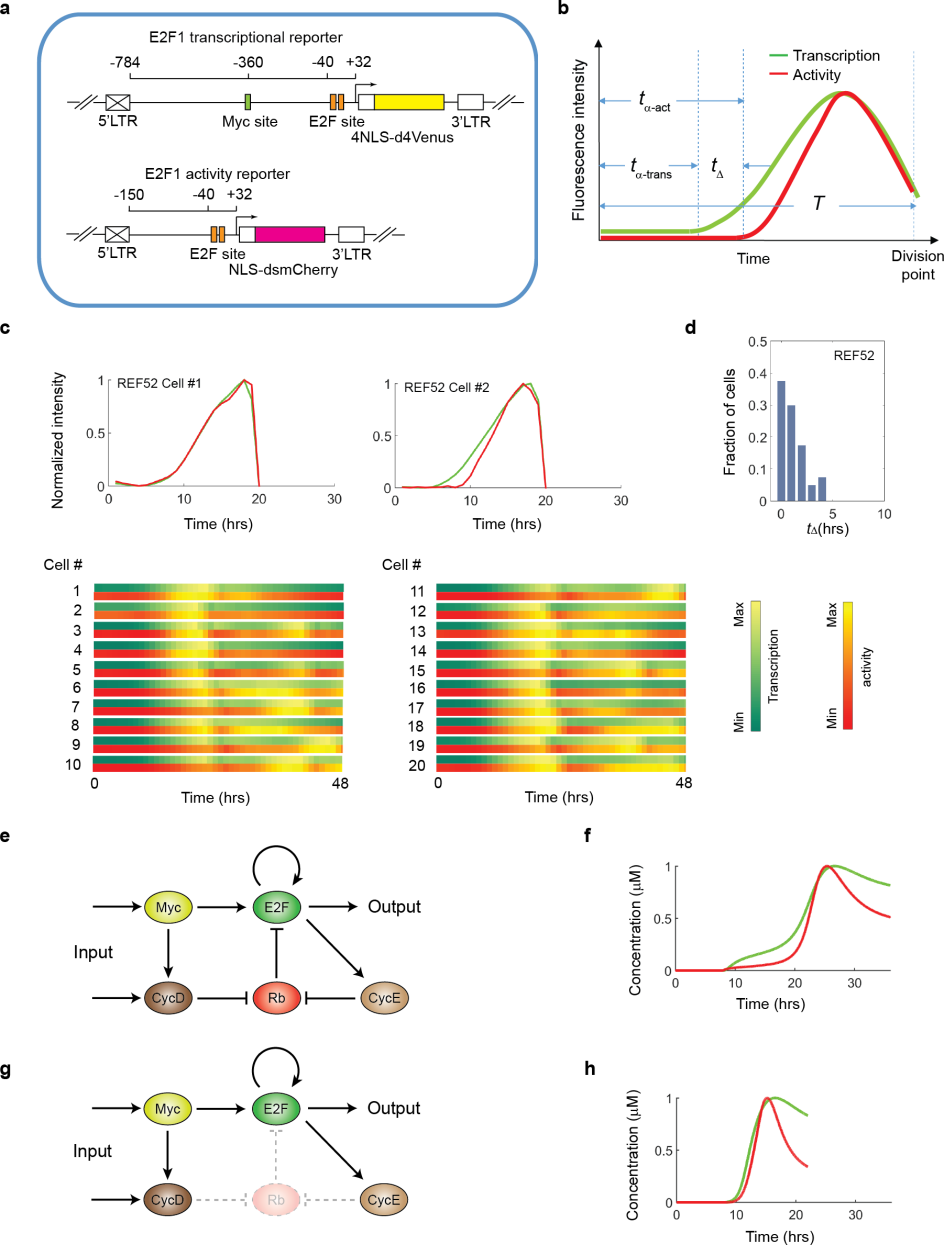
Origin and modulation of time delay between the onset of E2F1 transcriptional dynamics and that of E2F1 activity dynamics. (a) Schematic of the dual E2F1 reporter constructs. (b) Metrics to quantify the two types of dynamics: *t*_*α*-trans_: time before the onset of activation measured by the transcriptional reporter; *t*_α-act_: time before onset of activation measured by the activity reporter; *t*_Δ_ = *t*_α-act_ - *t*_*α*-trans_. *T*: duration of the whole cell cycle. (c) Representative trajectories of the E2F transcriptional dynamics and activity dynamics in individual cells released from serum starvation back into the cell cycle after addition of 10% BGS. Quantification of trajectory curves are shown in upper panels and 20 trajectories (48 hours) of both transcriptional (summer range colors and activity (autumn range colors) dynamics are shown below in color-coded bars. (d) Statistics of *t*_*Δ*_ between the two dynamics trajectories measured over ~50 cells under the condition of (c). (e) The Myc/Rb/E2F network. (f) Simulated trajectories of E2F transcriptional (green) and activity (red) dynamics. (g) The Myc/Rb/E2F network in which Rb is eliminated. (h) Simulated trajectories of E2F transcriptional (green) and activity (red) dynamics under conditions where Rb is eliminated.

In a previous study, we engineered a transcriptional reporter (relying on a Venus fluorescence signal) that captured E2F transcriptional dynamics at the single-cell level [19]. Since the E2F transcriptional dynamics is also characterized by an initial delay followed by a pulse of fluorescent signal–as the cell transits through the cell cycle–we decided to look more closely at how transcriptional and activity dynamics might differ. To this end, we introduced the two reporters into REF52 cells and derived clonal populations expressing both (Fig 3A). E2F transcriptional and activity dynamics were monitored in single cells during the first cell cycle after release into the cell cycle of cells kept in G0 by serum starvation. Alignment of the two trajectories shows that the activity signal always increases after the transcriptional signal is detected in any given cell (Fig 3B and 3C, S6 Fig). This lag-time (*t*_*Δ*_) varies substantially among individual cells (Fig 3D).

Given the Myc/Rb/E2F network structure (Fig 3E), it is not surprising that a delay exists between E2F transcriptional and activity dynamics, as (at least initially) newly synthesized E2F proteins would be immediately sequestrated by Rb. This delay is also predicted from our mathematical model of the Myc/Rb/E2F network, as time-course simulations run with the full model (S1, S3, S4 and S5 Tables) generated E2F transcriptional and activity dynamics with a temporal delay between the two trajectories (Fig 3F), consistent with our experimental observations. If the Rb node is removed, simulations show temporally overlapping trajectories of E2F transcriptional and activity dynamics (S2 Table, Fig 3G and 3H), confirming the role of Rb in generating *t*_*Δ*._

### Cyclin D/CDK4/6 activity controls *t*_*Δ*_ and G1 lengthening

**Fig 4:**
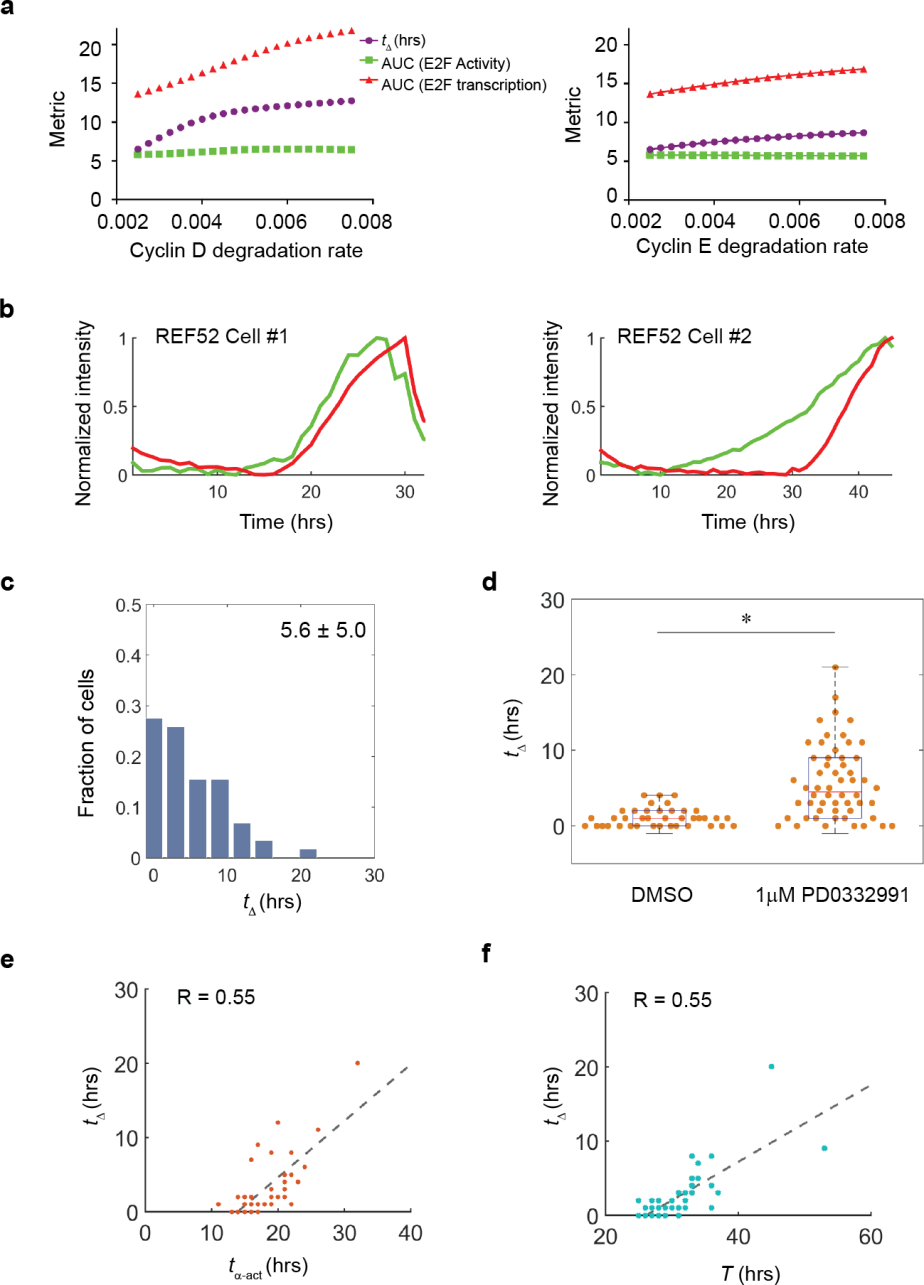
**Cyclin D/CDK4/6 activity controls the lag and G1 lengthening** (a) Simulation indicates prolonged *t*_*Δ*_ upon cyclin D/CDK4/6 and cyclin E/CDK2inhibition. (b) Individual trajectories of E2F transcriptional and activity dynamics in different cells released from serum starvation and entering cell cycle progression under condition of cyclin D/CDK4/6 inhibition. (c) Statistics of *t*_Δ_ between two dynamics trajectories over ~60 cells under the condition of (b). (d) Beeswarm and boxplot comparing *t*_*Δ*_ between normal condition and the condition of (b). *, p < 0.001. (e) Scatter plot of ~50 cells between *t*_*α*-act_ and *t*_Δ_. The individual dots were fitted in a linear relationship with R = 0.55. (f) Scatter plot of ~50 cells between *T* and *t*_*Δ*_. The individual dots were fitted in a linear relationship with R = 0.55.

Because Cyclin D/CDK4/6 complex is the primary regulator of Rb phosphorylation and functions prior to the action of cyclin E/CDK2, we reason that cyclin D/CDK4/6 complex inhibition will have dramatic effect on controlling *t*_*Δ*_ and G1 lengthening. Experimentally we treated REF52 cells expressing both the transcriptional and activity reporter with the CDK4/6-specific inhibitor–PD0332991. In concert with our prediction, alignment of both dynamics trajectories in individual cells over time indicates a significant increase of *t*_*α*-act_ under the inhibition of cyclin D/CDK4/6 activity (Fig 4B–4D).

Although the G1 phase duration shows the most variation among the different phases of the cell cycle, the origin of this variability remains poorly understood. Data obtained with the dual E2F reporter system suggest that G1 length (equal to *t*_*α*-act_) could be split into two periods, the first corresponding to an initial delay in the transcriptional dynamics (*t*_α-trans_) and the second being the delay between the initial increase in E2F activity and E2F transcriptional activation or *t*_Δ_ (Fig 3B). To investigate the contribution of *t*_*Δ*_ to the variance of the G1 length, we plotted *t*_*Δ*_ vs. *t*_*α*-act_ with each variance for the control group and groups under different inhibitor concentrations. Our results indicate that *t*_α-act_ increases roughly in proportion to the increase of *t*_Δ_, suggesting that *t*_*Δ*_ contributes primarily to the variation of G1 duration (Fig 4E). The linear relationship also holds when *t*_*Δ*_ is plotted vs. the whole cell cycle duration (*T*), suggesting that *t*_*Δ*_ is the predominant interval that influences the whole cell cycle duration (Fig 4F). Altogether, our findings suggest a model where the activity of the CycD/CDK4/6 complex, responsible for mono-phosphorylation of Rb is the main contributor in modulating the extent of the interval *t*_*Δ*_, and controlling G1 length during the cell cycle duration.

## Conclusions

E2F activity is regulated at different levels, including transcription, protein stability and Rb sequestration. In this study, we show that E2F activity (free E2F concentration) is constrained by E2F transcription but is modulated at the temporal scale through G1 cyclin/CDKs-mediated Rb phosphorylation. Taken together these studies provide a quantitative framework to describe the regulation of E2F activation during G1/S transition.

The information on both E2F transcriptional and activity dynamics provides a novel interpretation about the function of G1 cyclin/CDKs that regulate cell cycle commitment and progression. The prevailing view claims that the cyclin D/CDK4/6 activity is the trigger of cell cycle entry, whereas our results suggest that the cyclin D/CDK4/6 complex only control the timing of the onset of the E2F1 activity, even after E2F1 has been transcribed. This timing control reconciles the diverse, sometimes conflicting roles of the cyclin D/CDK4/6 activity in cell cycle regulation. First, cyclin D/CDK4/6 activity has been implicated as the driver of G1/S transition, because microinjection of anti-cyclin D1 antibody into normal diploid human fibroblasts prevented cells from entering S phase [26]. According to our single-cell analysis, however, the observed “prevention” of S phase entry might represent a marked postponement due to very slow release of E2F factors from Rb sequestration. A drastic delay caused by cyclin D/CDK4/6 inhibition might have been overlooked by conventional snapshot analyses of cell population exhibiting heterogeneous cycling time. Second, consistent with our data, overexpression of cyclin D shortened the G1 length but failed to initialize cell cycle entry in the absence of cooperation with other factors such as Myc [27]. Third, cells with deletion of the three D-type cyclins were still able to enter cell cycle but did so with a delayed response [28,29]. These findings were harder to reconcile with the canonical view of cell cycle control, and the explanation at the time was to invoke a hypothetical “cyclin D-independent” mechanism in cell cycle progression. In contrast, the above observations are consistent with our framework where cyclins primarily control the timing of the onset of E2F1 activity. Lastly, considering the inherent inertia of system kinetics that prevent an abrupt reversal of cell-fate decision, a steady slowing down might represent a more economic strategy to pause cell cycle progression when cells respond to internal stress signals.

Our findings have implications for the understanding of cellular senescence, which was described as a state of “permanent cell cycle arrest” but was poorly distinguished from other non-proliferative states such as quiescence and terminal differentiation at the mechanistic level. From the model supported by our data, senescence is likely to be a state in which cells are trapped within a permanent cell cycle progression due to the potent inhibition of G1 cyclin/CDK activities. Indeed, evidence has been provided in several studies that treatment of epithelial cells *in vitro* or *in vivo* with cyclin D/CDK4/6 inhibitors results in cellular senescence and tumor suppression [30,31]. Together our results from single-cell analysis clearly define a to-the-point function of cyclin D/CDK4/6 complexes as a modulator of G1 duration.

Cell cycle lengthening, particularly the extension of G1 phase, is a hallmark of cell cycle alteration during lineage specification in different physiological contexts [5,7]. Unraveling the control mechanism(s) mediating this lengthening may provide insights in our understanding of cellular differentiation and developmental processes. Since D-type cyclins are expressed in most cell types, including fibroblasts, epithelial cells, and various lineage progenitor cells [32]. Our proposed function for cyclin D/CDK4/6 as a master regulator of G1 timing provides an opportunity for future research in the field. Of particular interest will be to measure the expression of cyclin D/CDKs activity and correlate such activity with the speed of the cell cycle in various cell types. Such measurements will have to be done at the single-cell level considering the heterogeneity of cell cycle progression within a population. Moreover, regarding the critical role of cyclin D/CDKs activity in controlling cell cycle pace, it will be important to investigate how the activity dynamics is shaped by various upstream signaling pathways such as MAPK, Wnt and TGF-ß pathways and linked to cell fate decisions within different physiological contexts. Answers to these questions will clarify how cell cycle regulation affects normal proliferation, differentiation and senescence and how its deregulation can lead to pathological conditions such as cancer.

## Supporting information

S1 Fig Trajectories of E2F1 activity in single cells. REF52 cells (a) and hTert-HME1 cells (b) released from serum starvation. The dot in each trajectory corresponds to the cell division time point. Grey trajectories represent cells that did not divide during the observation window.
(TIF)

S2 Fig The distribution of AUC over around 70 cells. (TIF)

S3 Fig Reporter activity signals and Rb hyperphosphorylation in h-Tert-HME cells. Representative views of hTert-HME1 cells imaged in phase, mCherry and Alexa Fluo 488 (phosphorylated Rb) channels. Scale bar, 50 μm.
(TIF)

S4 Fig Reporter activity signals and EdU incorporation in h-Tert-HME cells. Representative views of hTert-HME1 cells imaged in phase, mCherry and Alexa Fluo 488 (EdU staining) channels. Scale bar, 50 μm.
(TIF)

S5 Fig Time delay between E2F activity onset and GFP-PCNA fine puncta staining. REF52 cells expressing the E2F activity reporter and a GFP-PCNA fusion protein were starved for 48h and released into the cell cycle with 10%BGS (t_0_). Cells were imaged every 30 min from t_0_ to t_60_ in the GFP and RFP channels (Olympus VivaView incubator microscope; 40X). (a) Time series of images (from time t_26_ to t_36_) showing the nuclear pattern of GFP-PCNA in a single cell. (b) The signal from the E2F activity reporter in cell shown in (a) was quantified. Activity values (relative fluorescent units) are reported from t_25_ (basal signal) to t_36_ (one frame before nuclear envelope breakdown). Arrow indicates time (t_28_) of first recorded increase in reporter activity over base line. (c) 47 single cells were scored for the delay between the onset of E2F activity and GFP-PCNA fine puncta formation. Delays fell into 5 categories (0–30 min; 30–60 min; 60–90 min; 90–120 min; 120–150 min). Counts indicate the number of cells in each time delay category.
(TIFF)

S6 Fig Origin and modulation of time delay between the onset of E2F1 transcriptional dynamics and that of E2F1 activity dynamics in hTert-HME1 cells. (a) Example trajectories of the E2F transcriptional dynamics and activity dynamics in cells released from serum starvation back into the cell cycle after growth stimuli. (b) Statistics of *t*_*Δ*_ between the two dynamics trajectories measured over ~50 cells under the condition of (a).
(TIF)

S1 Table Equations for the ODE model of Myc/Rb/E2F network. (DOCX)

S2 Table Equations for the ODE model of Myc/ E2F (Rb deleted) network. (DOCX)

S3 Table Values of model parameters. (DOCX)

S4 Table Variable definitions used for Myc/Rb/E2F network simulation analysis. (DOCX)

S5 Table Description of reaction terms. (DOCX)

S1 Movie Time-lapse movie showing E2F1 activity dynamics in REF52 cells. (AVI)

S2 Movie Time-lapse movie showing the variation of both E2F1 activity dynamics (red) and the signal of FUCCI Geminin-GFP reporter (S phase entry marker, green). (AVI)
