# Supplementary figures and images for: Cyclin D/CDK4/6 activity controls G1 length in mammalian cells
Source: PLoS One. 2018 Jan 8;13(1):e0185637. doi: 10.1371/journal.pone.0185637 (PMC5757913; doi:10.1371/journal.pone.0185637)

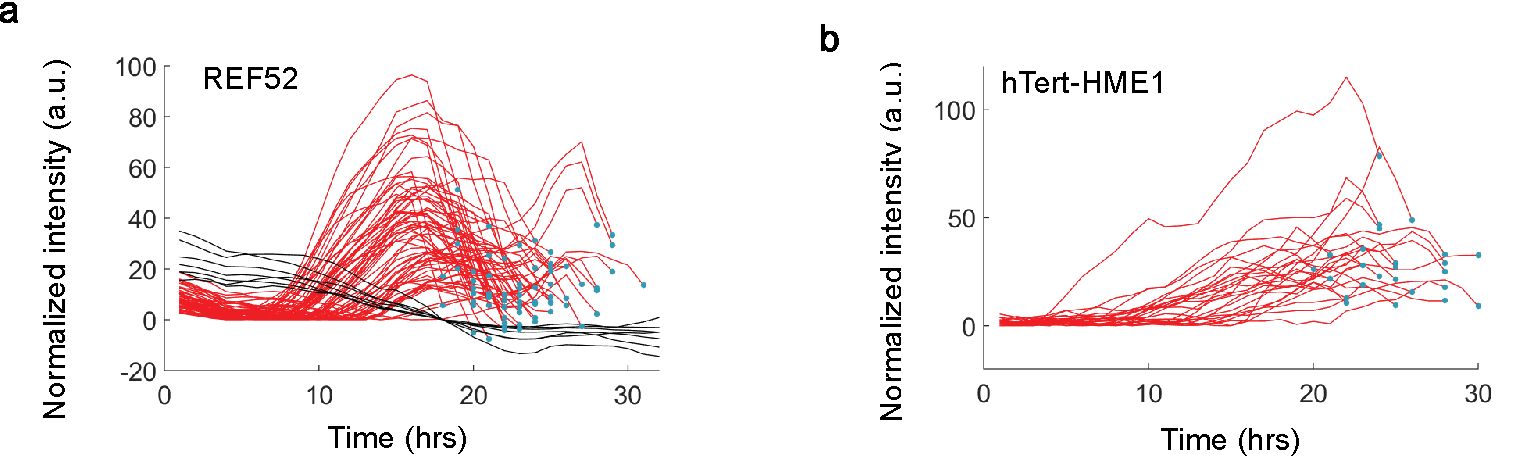

Supplement: S1 Fig — REF52 cells (a) and hTert-HME1 cells (b) released from serum starvation. The dot in each trajectory corresponds to the cell division time point. Grey trajectories represent cells that did not divide during the observation window. (TIF) [file pone.0185637.s001.tif]

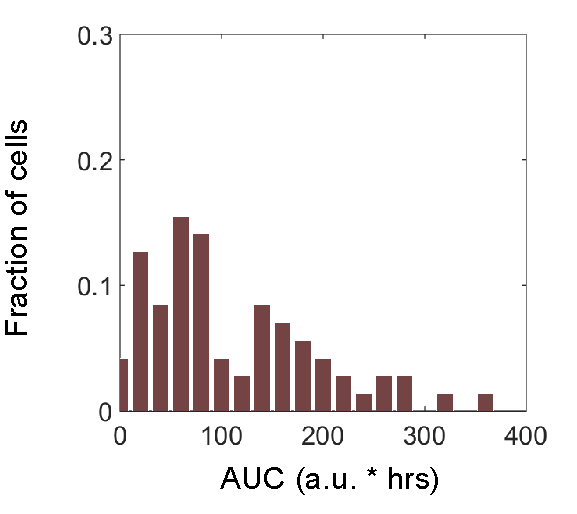

Supplement: S2 Fig — (TIF) [file pone.0185637.s002.tif]

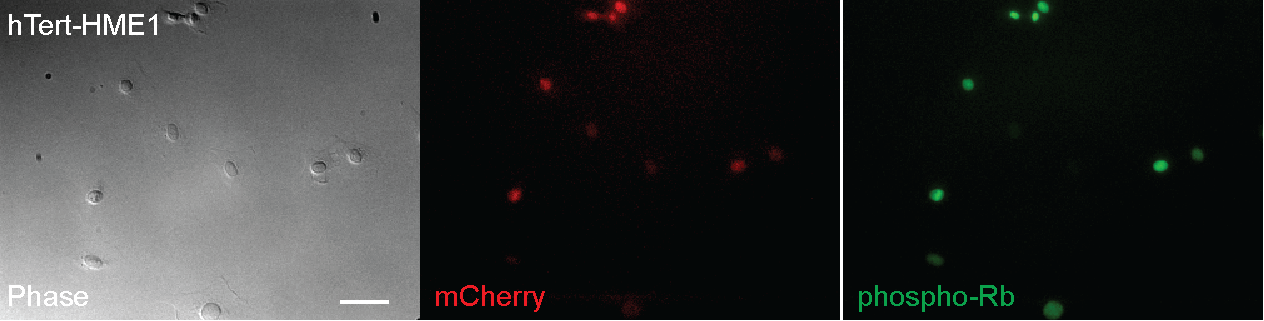

Supplement: S3 Fig — Representative views of hTert-HME1 cells imaged in phase, mCherry and Alexa Fluo 488 (phosphorylated Rb) channels. Scale bar, 50 μm. (TIF) [file pone.0185637.s003.tif]

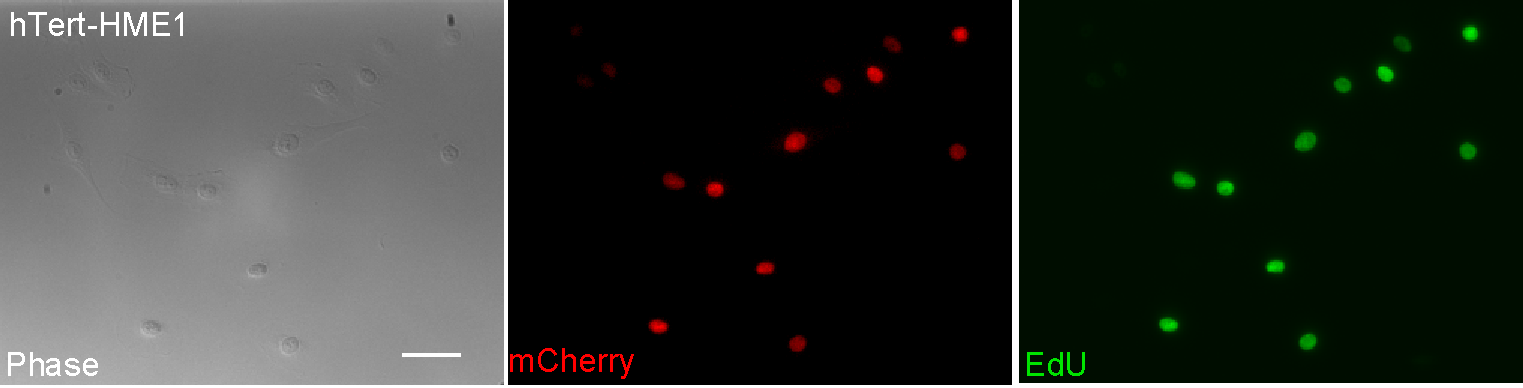

Supplement: S4 Fig — Representative views of hTert-HME1 cells imaged in phase, mCherry and Alexa Fluo 488 (EdU staining) channels. Scale bar, 50 μm. (TIF) [file pone.0185637.s004.tif]

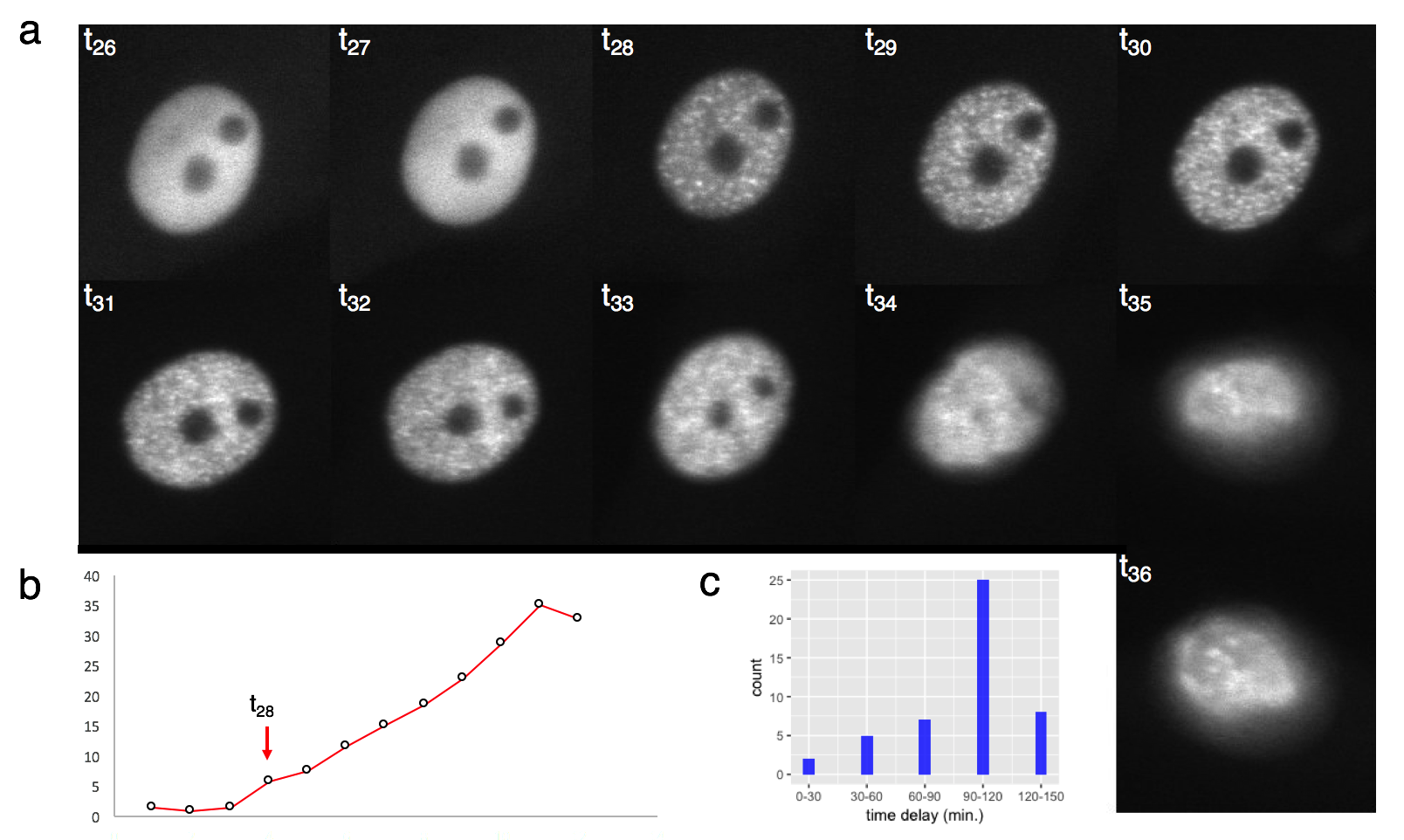

Supplement: S5 Fig — REF52 cells expressing the E2F activity reporter and a GFP-PCNA fusion protein were starved for 48h and released into the cell cycle with 10%BGS (t0). Cells were imaged every 30 min from t0 to t60 in the GFP and RFP channels (Olympus VivaView incubator microscope; 40X). (a) Time series of images (from time t26 to t36) showing the nuclear pattern of GFP-PCNA in a single cell. (b) The signal from the E2F activity reporter in cell shown in (a) was quantified. Activity values (relative fluorescent units) are reported from t25 (basal signal) to t36 (one frame before nuclear envelope breakdown). Arrow indicates time (t28) of first recorded increase in reporter activity over base line. (c) 47 single cells were scored for the delay between the onset of E2F activity and GFP-PCNA fine puncta formation. Delays fell into 5 categories (0–30 min; 30–60 min; 60–90 min; 90–120 min; 120–150 min). Counts indicate the number of cells in each time delay category. (TIFF) [file pone.0185637.s005.tiff]

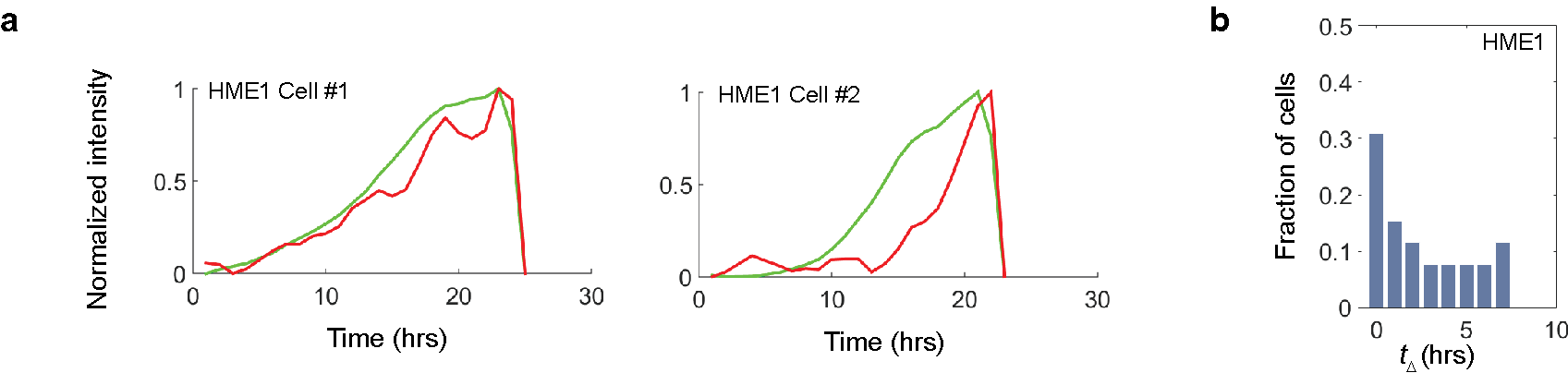

Supplement: S6 Fig — (a) Example trajectories of the E2F transcriptional dynamics and activity dynamics in cells released from serum starvation back into the cell cycle after growth stimuli. (b) Statistics of tΔ between the two dynamics trajectories measured over ~50 cells under the condition of (a). (TIF) [file pone.0185637.s006.tif]
