# Supplementary material for: Cyclin D/CDK4/6 activity controls G1 length in mammalian cells
Source: PLoS One. 2018 Jan 8;13(1):e0185637. doi: 10.1371/journal.pone.0185637 (PMC5757913; doi:10.1371/journal.pone.0185637)
Supplement: S2 Table — (DOCX) [file pone.0185637.s008.docx]

|  |
| --- |
|  |
|  |
|  |
|  |
|  |
