# Supplementary material for: Cyclin D/CDK4/6 activity controls G1 length in mammalian cells
Source: PLoS One. 2018 Jan 8;13(1):e0185637. doi: 10.1371/journal.pone.0185637 (PMC5757913; doi:10.1371/journal.pone.0185637)
Supplement: S3 Table — (DOCX) [file pone.0185637.s009.docx]

| **Parameter** | **Base Value** | **Description** |
| --- | --- | --- |
| $k_{\text{MC}}$ | 1 μM/h | MYC synthesis rate (by serum) |
| $d_{\text{MC}}$ | 0.7 /h | MYC decay constant |
| $k_{\text{b}}$ | 0.15 μM/h | E2Fm synthesis rate (by Myc alone) |
| $k_{\text{CD}}$ | 0.03 μM/h | Cyclin D synthesis rate (by Myc) |
| $k_{\text{CDS}}$ | 0.45 μM/h | Cyclin D synthesis rate (by serum) |
| $d_{\text{CD}}$ | 1.5 /h | Cyclin D decay constant |
| $k_{\text{RBP1}}$ | 18 /h | RB phosphorylation rate (by Cyclin D/CDK4/6) |
| $k_{\text{E2Fm}}$ | 0.40 μM/h | E2Fm synthesis rate (Myc/E2F co-regulation) |
| $d_{\text{E2Fm}}$ | 0.25 /h | E2Fm decay constant |
| $k_{\text{E2Fp}}$ | 0.40 /h | E2Fp translation rate |
| $d_{\text{E2Fp}}$ | 0.35 /h | E2Fp decay constant |
| $k_{\text{RBP2}}$ | 18 /h | Rb phosphorylation rate (by Cyclin E/CDK2) |
| $k_{\text{CE}}$ | 0.35 μM/h | Cyclin E synthesis rate (by E2Fp) |
| $d_{\text{CE}}$ | 1.5 /h | Cyclin E decay constant |
| $k_{\text{R}}$ | 0.10 μM/h | Repressor synthesis rate |
| $d_{\text{R}}$ | 0.10/h | Repressor decay constant |
| $k_{\text{RB}}$ | 0.18 μM/h | Rb synthesis rate |
| $d_{\text{RB}}$ | 0.06 /h | Rb decay constant |
| $k_{\text{RE}}$ | 18 /(μM*h) | Rb-E2F complex formation rate |
| $d_{\text{RE}}$ | 0.03 /h | Rb-E2F complex decay constant |
| $k_{\text{RBDP}}$ | 5 μM/h | RP dephosphorylation rate |
| $d_{\text{RP}}$ | 0.06 /h | RP decay constant |
| $K_{\text{S}}$ | 0.5 | Half-maximal serum concentration |
| $K_{\text{MC}}$ | 0.15 μM | Half-maximal Myc (E2Fm autoregulation) |
| $K_{\text{MCI}}$ | 2.5 μM | Half-maximal Myc (E2Fp-independent E2Fm regulation) |
| $K_{\text{MCCD}}$ | 0.15 μM | Half-maximal Myc (Cyclin D synthesis) |
| $K_{\text{EF}}$ | 0.15 μM | Half-maximal E2Fp (E2F autoregulation) |
| $K_{\text{CD}}$ | 0.92 μM | Half-maximal Cyclin D (Rb phosphorylation) |
| $K_{\text{CE}}$ | 0.92 μM | Half-maximal Cyclin E (Rb phosphorylation) |
| $K_{\text{RP}}$ | 0.01 μM | Michaelis-Menten constant (Rb dephosphorylation) |
| $K_{\text{R}}$ | 0.10 μM | Half-maximal R for E2Fm repression |
| $K_{\text{RS}}$ | 0.15 μM | Half-maximal E2Fp (R synthesis) |
