# Supplementary material for: Cyclin D/CDK4/6 activity controls G1 length in mammalian cells
Source: PLoS One. 2018 Jan 8;13(1):e0185637. doi: 10.1371/journal.pone.0185637 (PMC5757913; doi:10.1371/journal.pone.0185637)
Supplement: S4 Table — (DOCX) [file pone.0185637.s010.docx]

| S | Serum and mitogenic stimultion |
| --- | --- |
| MYC | Myc |
| E2Fm | E2F mRNA |
| E2Fp | Free E2F protein (not binding to Rb) |
| CD | Cyclin D |
| CE | Cyclin E |
| RB | Rb pocket protein |
| RE | Rb-E2F complex |
| RP | Phosphorylated RB |
| R | Repressor mediating E2F negative feedback loop  (*e.g.* Cyclin A and Skp2) |
