# Supplementary material for: Cyclin D/CDK4/6 activity controls G1 length in mammalian cells
Source: PLoS One. 2018 Jan 8;13(1):e0185637. doi: 10.1371/journal.pone.0185637 (PMC5757913; doi:10.1371/journal.pone.0185637)
Supplement: S5 Table — (DOCX) [file pone.0185637.s011.docx]

| **Reaction** | **Term** | **Description** |
| --- | --- | --- |
|  | $k_{\text{MC}}\cdot\frac{[S]}{K_{\text{S}}+[S]}$ | Serum-dependent Myc production |
|  | $d_{\text{MC}}\cdot[MYC]$ | MYC decay |
|  | $k_{\text{b}}\cdot\frac{[MYC]}{K_{\text{MCI}}+[MYC]}$ | E2Fm synthesis regulated by Myc alone |
|  | $k_{\text{E2Fm}}\cdot\frac{[MYC]}{K_{\text{MC}}+[MYC]}\cdot\frac{[E2Fp]}{K_{\text{EF}}+[E2Fp]}$ | E2Fm synthesis regulated by Myc/E2F cooperation |
|  | $d_{\text{E2Fm}}\cdot[E2Fm]$ | E2Fm decay |
| $\text{E2Fm}\to\text{E2Fp}$ | $k_{\text{E2Fp}}\cdot[E2Fm]$ | E2Fp production through translation |
| $\text{E2Fp+RB}\to\text{RE}$ | $k_{\text{RE}}\cdot[RB]\cdot[E2Fp]$ | Rb-E2F complex formation |
|  | $\frac{K_{\text{R}}+[R]}{K_{\text{R}}}\cdot d_{\text{E2Fp}}\cdot[E2Fp]$ | R-regulated E2Fp decay |
|  | $k_{\text{CD}}\cdot\frac{[MYC]}{K_{\text{MCCD}}+[MYC]}$ | Myc-dependent Cyclin D production |
|  | $k_{\text{CDS}}\cdot\frac{[S]}{K_{\text{S}}+[S]}$ | Serum-dependent Cyclin D production |
|  | $d_{\text{CD}}\cdot[CD]$ | Cyclin D decay |
|  | $k_{\text{CE}}\cdot\frac{[E2Fp]}{K_{\text{EF}}+[E2Fp]}$ | E2F-dependent Cyclin E production |
|  | $d_{\text{CE}}\cdot[CE]$ | Cyclin E decay |
|  | $k_{\text{RB}}$ | Constitutive Rb synthesis |
| $\text{RP}\to\text{RB}$ | $k_{\text{RBDP}}\cdot\frac{[RP]}{K_{\text{RP}}+[RP]}$ | Rb production through de-phosphorylation of RP |
|  | $k_{\text{RBP1}}\cdot\frac{[CD]\cdot[RB]}{K_{\text{CD}}+[RB]}$ | Cyclin D-dependent phosphorylation of Rb |
|  | $k_{\text{RBP2}}\cdot\frac{[CE]\cdot[RB]}{K_{\text{CE}}+[RB]}$ | Cyclin E-dependent phosphorylation of Rb |
|  | $k_{\text{RBP1}}\cdot\frac{[CD]\cdot[RE]}{K_{\text{CD}}+[RE]}$ | E2Fp release due to Cyclin D-dependent phosphorylation of RE |
|  | $k_{\text{RBP2}}\cdot\frac{[CE]\cdot[RE]}{K_{\text{CE}}+[RE]}$ | E2Fp release due to Cyclin E-dependent phosphorylation of RE |
|  | $d_{\text{RB}}\cdot[RB]$ | Rb decay |
|  | $d_{\text{RP}}\cdot[RP]$ | RP decay |
|  | $d_{\text{RE}}\cdot[RE]$ $k_{\text{R}}\cdot\frac{[E2Fp]}{K_{\text{R}}+[E2Fp]}$ | RE decay |
|  | $k_{\text{R}}\cdot\frac{[E2Fp]}{K_{\text{R}}+[E2Fp]}$ | E2F-dependent R transcription |
|  | $d_{\text{R}}\cdot[R]$ | R decay |
